# A Causal Theory of Mnemonic Confabulation

**DOI:** 10.3389/fpsyg.2017.01207

**Published:** 2017-07-18

**Authors:** Sven Bernecker

**Affiliations:** ^1^Department of Philosophy, University of California, Irvine Irvine, CA, United States; ^2^Department of Philosophy, University of Cologne Cologne, Germany

**Keywords:** false memory, forgetting, misremembering, relearning, knowledge, justification, counterfactuals

## Abstract

This paper attempts to answer the question of what defines mnemonic confabulation vis-à-vis genuine memory. The two extant accounts of mnemonic confabulation as “false memory” and as ill-grounded memory are shown to be problematic, for they cannot account for the possibility of veridical confabulation, ill-grounded memory, and well-grounded confabulation. This paper argues that the defining characteristic of mnemonic confabulation is that it lacks the appropriate causal history. In the confabulation case, there is no proper counterfactual dependence of the state of seeming to remember on the corresponding past representation.

Memory errors can be grouped into two categories: errors from omissions and errors from commission. Omissions are forgetting errors. Commissions are “false memories.” Confabulation is a kind of commission error that occurs when patients produce stories that fill in gaps in their memories^1^. Different neurological syndromes are known to cause confabulation. Among them are Korsakoff's syndrome (memory disorder caused by lack of vitamin B1 often due to chronic alcohol abuse), split-brain syndrome, anosognosia for hemiplegia (denial of paralysis), Anton's syndrome (denial of blindness), and Capgras syndrome (the illusion that an impostor has replaced a person close to the patient). Confabulation can also occur in healthy adults and young children. Children sometime offer narratives of fabricated events that are so plausible and convincing that even professional child psychologists cannot distinguish from proper memories (Ceci, 1995; Ackil and Zaragoza, 1998). Subjects under hypnosis may confabulate when they are asked to recall information (Dywan, 1995). Moreover, there are a number of experiments that suggest that we sometimes claim to remember having experienced events that we have only imagined (Ceci, 1995; Hyman et al., 1995).

What distinguishes confabulations from delusions? Some consider delusional beliefs to be a non-memorial form of confabulation (Coltheart and Turner, 2009), others consider delusions to be false or ill-grounded *beliefs* while confabulations are false or ill-grounded *claims* (Hirstein, 2005, p. 18), and yet others claim that delusions are *acceptances* as opposed to *beliefs* (Dub, 2017). Regardless of whether delusions are beliefs, claims, or acceptances, there are two main differences between confabulations and delusions. First, confabulations can be coherent with the rest of the subject's beliefs while delusions are typically not. The possible coherence of confabulations is discussed in Section “Against the Epistemic Theory of Confabulation”. Second, delusions, but not confabulations, are necessarily resistant to counterevidence and impervious to counterargument (Dub, 2017).

Needless to say, not all confabulations concern the past and not all of them arise from failures in the memory system. Confabulations may also occur within the perceptual and affective modules. That said, the primary focus of this paper is on mnemonic confabulation.

The American Psychiatric Association's *Diagnostic and Statistical Manual of Mental Disorders* (DSM for short), the “bible” for psychiatric diagnosis, does not explicitly define confabulation but states that confabulation is “often evidenced by the recitation of imaginary events to fill in gaps in memory” (The American Psychiatric Association, 1994, p. 157). According to this definition, the hallmark of confabulation is that it is fictitious. While falsity might be a sufficient condition for confabulation, it is not necessary. Not every “false memory” qualifies as confabulation. Another characteristic of confabulation, besides falsity, is that the subject is unaware that she is confabulating, and takes the “false memory” to be accurate. Most definitions of confabulation list falsity and the absence of deceptive intent as individually necessary and jointly sufficient conditions for confabulation. Consider the following definitions:

Confabulations are inaccurate or false narrative purporting to convey information about the world or self (Berrios, 2000, p. 348).

Confabulation is a symptom which is sometimes found in amnesic patients and consists in involuntary and unconscious production of “false memories,” that is the recollection of episodes, which never actually happened, or which occurred in a different temporal-spatial context to that being referred to by the patient (Dalla Barba, 2002, p. 28).

Confabulations are typically understood to represent instances of false beliefs: opinions about the world that are manifestly incorrect and yet are held by the patient to be true in spite of clearly presented evidence to the contrary (Turnbull et al., 2004, p. 6).

In the broad sense confabulations are usually defined as false narratives or statements about the world and/or self-due to some pathological mechanism or factors, but with no intention of lying (Örulv and Hydén, 2006, p. 648).

Confabulations are false memories produced without conscious knowledge of their falsehood (Fotopoulou, 2008, p. 543).

What all of these definitions have in common is the focus on epistemic surface features such as belief, truth, evidence, knowledge, intention. None of these definitions makes reference to the underlying mechanisms that are causally responsible for confabulation. The reason is that there are numerous neuropsychological conditions that can give rise to confabulation and that confabulation can also occur without a (known) neuropsychological deficit^2^.

The standard definition of confabulation as “false memory” produced without awareness of its falsity is problematic for three reasons: first, not all confabulations are wholly false; some are partially true and partially false. Second, it is possible that a confabulation is wholly true but still epistemically defective because it is true merely by accident. Third, according to some, genuine memories need not be completely accurate. Given that (episodic) remembering is an inherently constructive process, memory contents may be false to some degree. Given that confabulations may be true by luck and that genuine memories need not be wholly true, the standard definition fails to draw a strict line of demarcation between memories and confabulations.

In light of the problems with the standard definition of confabulation, some have proposed an epistemic definition of confabulation as ill-grounded or unjustified memory (cf. Hirstein, 2005, ch. 8; Michaelian, 2016b, p. 5–7). The epistemic account of confabulation promises to avoid the problems of the standard account by making room for veridical confabulation and for partially false memory. But the epistemic account too blurs the distinction genuine memory and confabulation, for there are genuine memories that are ill-grounded (unjustified) as well as properly justified confabulations.

This paper argues that what defines confabulation vis-à-vis genuine memory is not that it is false or ill-grounded but that it lacks the appropriate causal history. The hallmark of confabulations is that they fail to satisfy the causal condition on remembering.

To avoid misunderstandings, it is worth addressing a general worry that one might have about philosophical attempts to define medical terms like “confabulation.^3^” The fact that the DSM does not explicitly define confabulation and that the literature on the topic contains a number of different definitions can be taken to suggest that the medical profession is still in the process of coming up with a precise definition of the concept of confabulation. But if this is so then it seems premature for philosophy to try to define confabulation. Maybe philosophy should refrain from defining the term “confabulation” until after the medical sciences have clearly identified the phenomena denoted by it.

This paper is to be understood as a friendly offer of help to the medical sciences. Philosophy is, as James (1987, p. 296) noted, “an unusually stubborn effort to think clearly.” As such philosophy is well-positioned to offer novel perspectives on issues in psychiatric classification (see Sadler et al., 1994; Perring, 2010). I would be pleased if the causal theory of confabulation developed in this paper proves to be a useful contribution to the ongoing pursuit to come up with a medical definition of confabulation that is both precise and exhaustive.

Section “The Constructive Nature of Memory” argues that a memory state must be factual in the sense of accurately representing the objective reality and “authentic” in the sense of resembling the subject's initial perception of reality. Section “Confabulation without Falsehood” argues against the standard conception of confabulation as “false memory” by pointing out that confabulations may be veridical. Section “Against the Epistemic Theory of Confabulation” criticizes the epistemic conception of confabulation by pointing out that there are properly grounded (justified) confabulations. Section “The Causal Theory of Memory” explains the causal condition on remembering and Section “A Causal Theory of Mnemonic Confabulation” argues that the distinctive feature of confabulations is that they fail to meet the causal condition. Section “Confabulating, Misremembering, and Relearning” discusses the clinical utility of the causal theory of mnemonic confabulation vis-à-vis some of its competitors.

## The constructive nature of memory

According to the standard conception of memory in philosophy, propositional memory works like a photocopier producing duplicates of past propositional attitudes^4^. The content of a memory state must be type-identical to the content of the original propositional attitude from which it causally derives. This *xerox model* of memory goes back to antiquity. In the *Theaetetus*, Plato (1921, 191c8-e) compares (phenomenal) memory to a block of wax in which the perceptions are imprinted in the same way “as we might stamp the impression of a seal ring. Whatever is rubbed out or has not succeeded in leaving an impression we have forgotten and so do not know.” The wax tablet metaphor is taken up by Aristotle (1972) in *De Memoria* (450a, p. 28–32)^5^. Augustine (1991, p. 191) calls the memory the “belly of the mind” and compares it to “a large and boundless inner hall,” a “storehouse,” and a “vast cave” within which “the images of things perceived” are laid away, to be “brought forth when there is need for them.” Similarly, Hume (2000, p. 12) maintains that memory is about the re-experiencing of mental images that are copies of the original experience. He goes so far as to claim that “memory preserves the original form, in which its objects were presented, and that wherever we depart from it in recollecting anything, it proceeds from some defect or imperfection in that faculty.”

The xerox model of memory is at odds with what science tells us about the workings of memory. Instead of being etched in a wax-tablet-like stable form long-term memories are retained by a miniature molecular process that must run constantly to maintain the memories; jamming this process can erase long-term memories (cf. Shema et al., 2007). Furthermore, it has been shown that memory is not only a passive device for reproducing contents but also an active device for processing stored contents. The psychologist Engel explains:

One creates the memory at the moment one needs it, rather than merely pulling out an intact item, image, or story. This suggests that each time we say or imagine something from our past we are putting it together from bits and pieces that may have, until now, been stored separately. Herein lies the reason why it is the rule rather than the exception for people to change, add, and delete things from a remembered event (Engel, 1999, p. 6).

Given the constructive nature of retrieval in memory, the question arises as to what extent two propositional attitude tokens may be different from one another and one of them still is memory-related to the other. If remembering does not require the duplication of past propositional attitudes, what is the permissible range of aberration between a propositional attitude and the memory thereof? To answer this question, it is useful to group the ways in which our memory processes contents into two categories: mnemonic processes that, when working properly, preserve the truth-value of the encoded content and mnemonic processes that change the truth-values of the encoded contents^6^. Examples of mnemonic processes of the former kind are leveling, cognitive dynamics, and boundary extension. Leveling refers to the loss of details, the condensation of elements, and the general simplification of the information encoded in memory^7^. Leveling preserves the truth of the encoded content, for, say, if it is true that there is a blue jay sitting in the mulberry tree, then it is true that there is a bird sitting in the tree^7^. “Cognitive dynamics” is Kaplan's (1989) term for the ability to vary a judgment so as to manifest a single persisting belief. When you keep track of someone or something as you (or both) of you move or as time passes by, you must vary the indexicals and tensed verbs in a judgment so as still to express the same belief as before. Boundary extension is the phenomenon in which a subject claims to remember seeing a surrounding region of a scene that was not visible in the studied view. Although boundary extension is an error of commission with respect to the stimulus (and thus violates the authenticity condition discussed below), it is more often than not a reliable prediction of the world that did exist just beyond the edges of the subject's original view^9^.

Some mnemonic processes, even when working properly, change the truth-values of the encoded contents. Cases in point are misattribution, suggestibility, and confabulation. Misattribution involves assigning a memory to the wrong source: mistaking fantasy for reality, or incorrectly remembering that, say, a friend told you a bit of trivia that you actually read about in a newspaper. Suggestibility refers to memories that are implanted as a result of leading questions, comments, or suggestions when a subject is trying to remember something.

The question has been raised before as to what extent two propositional attitude tokens may be different from one another and one of them still be memory-related to the other. Elsewhere Bernecker (2010, p. 36–9, 213–239) I argue that for a mental state to qualify as a memory it must accurately represent the objective reality *and* resemble the subject's initial perception of reality. Memories must be *veridical* in the sense of being factually correct and they must be *authentic* in the sense of accurately reflecting the subject's past viewpoint. A memory counts as authentic if it stems from a truth-preserving process such as leveling, cognitive dynamics, and boundary extension (On this view, one can fail to remember something not only because there is something wrong with one's memory but also because the representation fed into the memory process is false. Memory neither allows for a mistake of inheritance nor for the inheritance of a mistake.).

In Bernecker (2010, ch. 8) I argue that non-inferential remembering-that allows for the contents of the past and present representations not to be type-identical but only sufficiently similar. A memory content is sufficiently similar to the content of one's prior mental state so as to count as an instance of non-inferential remembering (provided all the other memory conditions are met) if it must be either identical to, or relevantly entailed by, the original content^10^. The so-called *entailment thesis* is perfectly compatible with the veridicality constraint on remembering. The reason is that the entailment relation preserves truth. If the contents fed into the memory process are veridical and there are no external circumstances changing the truth-values of the contents while they are in storage, the entailment thesis ensures that the retrieved contents are veridical as well. And since each proposition entails itself the entailment thesis also allows for cases where our memory works like a photocopier producing duplicates of past propositional attitudes.

The view whereby remembering requires both truth and authenticity is not shared by everyone in the field. Some drop the truth condition and merely require that memories be authentic (e.g., Newby and Ross, 1996). Others weaken the truth condition by maintaining that memories only need to contain some truths but need not be completely true. Bernstein and Loftus, for example, write:

All human memory is false to some degree. Memory is inherently a reconstructive process, whereby we piece together the past to form a coherent narrative that becomes our autobiography. In the process of reconstructing the past, we color and shape our life's experiences based on what we know about the world^11^.

Similarly, Conway and Loveday declare:

All memories are to some degree false in the sense that they do not represent past experience literally.…One of the main functions of memories is to generate meanings, personal meanings, that allow us to make sense of the world and operate on it adaptively. Memories are, perhaps, most important in supporting a wide range social interactions where coherence is predominant and correspondence often less central^12^.

To be sure, there is a crucial difference between saying, as I do, that memories must be veridical even though they need not amount to the exact reproduction of some previously recorded content and saying, as the above mentioned authors do, that memories need not be (completely) true. There is general agreement that the human memory is meant to not only store but also process the encoded information. As a result of such information processing, the content of the memory state may differ, to some degree, from the content of the original propositional attitude from which the memory causally derives. The dispute is about the degree to which memory may change the encoded contents. In what follows, I presuppose that a mental state qualifies as a memory only if it accurately represents the objective reality and accords with the subject's initial perception of reality.

## Confabulation without falsehood

The aim of this section is to challenge the standard definition of confabulation as “false memory” by arguing that confabulations may meet the authenticity constraint and that they may accurately represent the objective reality.

Different classification schemes for kinds of confabulation have been proposed in the literature. According to Schnider (2008, p. 63–64), there are four kinds of confabulation: (1) *Intrusions* in memory tests are occasional distortions when a subject is asked to recall the details of a story. (2) *Momentary confabulations* are false verbal statements in a discussion or another situation inciting a patient to make a comment. These confabulations are inherently plausible but frequently false. (3) *Fantastic confabulations* have no basis in reality and, unlike momentary confabulations, are inconceivable, non-sensical, and implausible. (4) *Behaviorally spontaneous confabulation* constitutes a syndrome composed of momentary and fantastic confabulation, amnesia, and disorientation. The hallmark of this type of confabulation is that the patients act according to their confabulations.

Fantastic and behaviorally spontaneous confabulations are patently false, self-contradictory, and bizarre. Intrusions and momentary confabulations, on the other hand, may be “coherent, internally consistent, and relatively commonplace” (Moscovitch, 1995, p. 226–227)^13^. These kinds of confabulations tend to be coherent not only at a particular time but over a longer period of time. It has frequently been observed that patients persist in their confabulations even in the face of evidence to the contrary^14^. And given that internal consistency is a crucial component of authenticity, it is not unreasonable to suppose that confabulations may satisfy the authenticity constraint characteristic of remembering.

Just as confabulations need not be inconsistent, they need not misrepresent the objective reality. It is conceivable that confabulations contain few or no falsehoods (Michaelian, 2016b, p. 4). Confabulations having to do with autobiographical information may reference actual states of affairs, but only miscontextualize them in time (Talland, 1965, p. 56; Dalla Barba et al., 1990). A mother may, for instance, rightly recollect that she has children, but not recall that they have grown up and left home. A piece of confabulation may even be entirely correct. It is possible that a patient fantasizes correctly by telling a story that, by sheer luck, represents the objective reality. A confabulatory hypochondriac, for instance, may seem to remember having had thyroid cancer with little or no evidence. The truth is, however, that he did have thyroid cancer but it was never detected. McKay and Kinsbourne imagine a subject who lacks “access to his biographical information, yet by chance may confabulate the correct answer when asked his age” (McKay and Kinsbourne, 2010, p. 289; cf. Berlyne, 1972, p. 32 cited in Hirstein, 2005, p. 9). The upshot is that it cannot be ruled out that confabulations meet not only the authenticity condition but also the veridicality condition of memory.

In sum, a confabulatory process can lead to the formation of either a true or a false representation. So while the notion of “false memory” is familiar enough but an oxymoron the notion of “veridical confabulation” is unfamiliar but denotes a possible situation.

## Against the epistemic theory of confabulation

In light of the problems facing the standard definition of confabulation as “false memory” some have proposed that the key feature of confabulations is that they are ill-grounded, poorly supported by evidence, or unjustified (I use “justification” to refer to that, whatever precisely it is, which together with truth makes the difference between knowledge and mere true belief. In this sense, reliability is a kind of justification.) The obvious advantage of such an *epistemic* account of confabulation over the standard account is that it can handle cases of veridical and consistent confabulation.

Whether the notion of confabulation as “false memory” is distinct from the notion of confabulation as unjustified memory depends on where one stands with respect to the debate between fallibilism and infallibilism about justification. Infallibilism is the view that a belief cannot be at once justified and false. Complete justification necessitates or entails truth. Infallibilism is a minority view because it is thought to lead to skepticism. Since only on rare occasions our evidence for some proposition guarantees that it is true, infallibilism seems to have the counterintuitive consequence that justification is a rare commodity. If we can know things only on the basis of deductive arguments, then very few of the things we ordinarily believe on the basis of inductive, perceptual or testimonial evidence qualify as knowledge. This is why most epistemologists endorse some version of fallibilism. According to fallibilism, it is possible that a belief is completely justified yet false and that a belief is true yet unjustified^15^. Given fallibilism, there is a marked difference between the standard account of confabulation as “false memory” and the epistemic account of confabulation as unjustified memory. In what follows, I assume fallibilism.

Bortolotti (2010, p. 44–45) motivates the epistemic notion of confabulation as follows:

What seems to be relevant to the detection of the phenomena of confabulation and delusion is not whether the reported state is true, but whether its content conflicts with other things the subject believes, or is held with a level of conviction that is not explained by its plausibility or the evidential support available for it.

The foremost advocates of the epistemic notion of confabulation are Hirstein (2005, ch. 8) and Michaelian (2016b, p. 5–7). Hirstein maintains that the hallmark of confabulation “is not the falsity itself, but that the claims are being produced by a malfunctioning cognitive system, which is producing ill-grounded thoughts.” Hirstein goes on to propose the following conceptual analysis: Jan confabulates that p if and only if

(i) Jan claims that p; (ii) Jan believes that p; (iii) Jan's thought that p is ill-grounded; (iv) Jan does not know that her thought is ill-grounded; (v) Jan should know that her thought is ill-grounded; (vi) Jan is confident that p (Hirstein, 2005, p. 187).

Michaelian's approach to confabulation is broadly in line with Hirstein's definition in terms of the notion of ill-groundedness. But while Hirstein's notion of justification combines internalist and externalist elements, Michaelian defines justification from an externalist-reliabilist perspective. He writes:

Confabulation…occurs when the subject's episodic memory system function unreliably. When the system functions unreliably, it will usually produce an inaccurate representation. In cases where an unreliably functioning memory system produced an inaccurate representation, the subject can be said to confabulate falsidically…In cases where an unreliably functioning memory system produces an accurate representation, the subject can be said to confabulate veridically (Michaelian, 2016b, p. 6).

There are three problems with the epistemic account of confabulation in general and with Hirstein's account in particular. Let us discuss these problems in turn.

The first problem concerns condition (iv) which is supposed to capture the idea that the confabulatory patient has no intention of lying^16^. As stated, condition (iv) is too weak since it only rules out cases where the confabulator does not *know* that her thought is ill-grounded. This condition is satisfied when the confabulator *believes* that her thought is ill-grounded but where she lacks sufficient reasons for her belief and hence does not qualify as *knowing* that her thought is ill-grounded. A subject may not know that her thought is ill-grounded because her belief to the effect that her thought is ill-grounded is not true, is not justified or because it is gettierized. A simple solution to th problem at hand is to strengthen condition (iv) as follows:

(iv^*^) Jan does not (justifiedly) believe that her thought is ill-grounded.

Instead of only requiring that the confabulator does not *know* that her thought is ill-grounded condition (iv^*^) demands that the confabulator does not even (justifiedly) believe that her thought is ill-grounded^17^. Condition (iv^*^) seems to be doing a better job of capturing the gist of what Hirstein has in mind.

Another problem with Hirstein's account of confabulation concerns condition (v) which states that Jan *should* know that her thought is ill-grounded: “if the confabulator's brain were functioning properly, she would know that the claim is ill-grounded and not make it” (Hirstein, 2009, p. 652)^18^. Given that ought implies can, condition (v) rules out cases where the subject is not in a position to know that her belief is ill-grounded because the grounds for the belief are not accessible via introspection and reflection. To illustrate this point, consider a much cited study by Nisbett and Wilson (1977) where normal subjects were asked to provide their reasons for selecting the best pair of nylon pantyhose from an array of identical pantyhose. Participants selected the pantyhose on the right and produced confabulated explanations referencing its particular features (the knit, elasticity, sheerness, etc.) rather than its position on the vending table. Speakers of Indo-European languages read from left to right and so tend to scan the field of vision from left to right. Whatever is on the far right is seen last and has a preeminent status in regard to choice. Yet the participants denied that their choice was influenced by position. The point is that the participants in the experiment had no obligation to know why they preferred the rightmost pantyhose because they lacked introspective access to the causes of their choices. This indicates that condition (v) is too stringent. Note that condition (v) would still be too stringent if it stated that the confabulator should believe (as opposed to know) that her thought is ill-grounded^19^.

Hyponotic suggestion provides another example of people reporting ill-grounded beliefs without being aware that their beliefs are ill-grounded. In an experiment conducted by Rahmanovic et al. (2012) and reported by Bortolotti and Cox (2009, p. 959) hypnotized participants received a suggestion that their non-dominant hand and arm belongs to someone else and they were instructed to forget the fact that the hypnotist gave them this suggestion. The hypnotized participants were then asked to pick up objects on a tray located next to the arm targeted by the suggestion. If they used the arm not targeted by the suggestion they were asked why they used this arm. They offered confabulated reasons such as “my other arm is stuck” or “my other arm is paralyzed.” The participants were also challenged by being asking what they would say if a doctor examined their arm and found that the arm was normal and that it belonged to the participant. Some of the participants commented that the doctor would be wrong. Since the participants in the experiment lacked awareness of why their arm felt differently they were not in a position to know or believe that their beliefs were ill-grounded. The upshot is once again that Hirstein's condition (v) is too stringent.

One might be tempted to handle ought-implies-can based objections to condition (v) by adding an “under normal conditions” clause. The revised condition then reads:

(v^*^) Jan should know under normal conditions that her thought is ill-grounded.

Yet the problem with condition (v^*^) is that there are many normal (non-pathological) situations where it is psychologically impossible for a subject to become aware of the ill-groundedness of her thoughts. Cases in point include implicit biases and stereotypes. Presumably Hirstein will want to exclude ordinary biases and stereotypes from the definition of confabulation. But then the “under normal conditions” phrase is tantamount to “in non-confabulatory situations” and the definition of confabulation turns out to be circular.

The third and most serious problem with the epistemic notion of confabulation concerns condition (iii) which characterizes confabulatory thoughts as ill-grounded or unjustified. Condition (iii), unlike condition (v), is an *essential* component of any epistemic account of confabulation. There are two kinds of counterexamples to the thesis that confabulations, but not memories, are ill-grounded: it is possible for genuine memories to be *unjustified* and for confabulations to be *justified*. The epistemic account therefore fails to differentiate between confabulation and memory.

The most compelling cases of memory without justification are ones where the subject remembers that p but where there is some defeating information such that, if the subject became aware of it, she would no longer be justified in believing p^20^. This is not the place to argue on behalf of the necessity of a no-defeater condition for justification. In addition to the sheer plausibility of the view that justification is incompatible with the presence of undefeated defeaters, the literature is dominated by endorsements of no-defeater conditions. Despite the great variety of conceptions of epistemic justification, philosophers on both sides of the internalism/externalism divide sign up to the idea that justification is incompatible with undefeated defeaters. In the case of epistemic internalism, it is obvious that the presence of undefeated defeaters undermines justification. For if what justifies a belief is a mentally accessible item (something that one can come to know whether it obtains just by reflecting on one's mental states), being justified in believing p must exclude a person's having sufficient reasons for supposing either that p is false or that the belief that p is not grounded or produced in a way that is sufficiently truth-indicating.

Whether the presence of undefeated defeaters is compatible with the externalist construal of justification depends on the version of externalism under consideration. Given an austere form of epistemic externalism, a subject is epistemically justified in believing something just in case the belief is truth-effective; it doesn't matter whether the subject takes his belief to be unjustified. As long as one relies on what is, in point of fact, a good reason for p, one is justified in believing that p, despite being convinced that p is false or despite being convinced that the belief that p is unreliably formed. This position is labeled “mad-dog reliabilism” in Dretske (2000, p. 595). For reasons I don't have space to go into here mad-dog reliabilism is generally rejected. All of the leading advocates of externalist reliabilism—Alvin Goldman, Robert Nozick, and Alvin Plantinga, to mention only a few—adopt no-defeater conditions. They hold that although a subject need not be aware of the factors that justify his belief, he may not be aware of evidence that undermines his belief. And there is no inconsistency in affirming that what confers justification on a belief is an externalist condition, but what takes justification away from a belief is an internalist no-defeater condition. The no-defeater condition ensures that for a belief to become justified it must not be incoherent with the background information the subject possesses.

In light of these preliminary points consider the following case of remembering without justification. At t_1_, Jill came to know that John F. Kennedy was assassinated in 1963^21^. At t_2_, Jill's friends play a practical joke on her. They tell her that Kennedy was assassinated in 1964 and present her with plausible but misleading evidence to this effect. Given the incompatibility of justification with the presence of undefeated defeaters, Jill doesn't know at t_2_ that Kennedy was assassinated in 1963. The reason she doesn't know this is because she is unable to rule out the relevant alternative that Kennedy was not assassinated until 1964. Jill fails to know that Kennedy was assassinated in 1963, despite the fact that she still remembers this fact from what she knew at t_1_. This example suggests that one can remember what one knew but doesn't know anymore—even though one continues to truly believe it—for the reason that one isn't any more fully justified in believing it^22^.

As mentioned above, there are two kinds of counterexamples to the thesis that confabulations, but not memories, are unjustified. The first kind of counterexamples concerned *unjustified* memories; the second kind concerns *justified* confabulations. Depending on how the notion of justification is construed, confabulations may qualify as being properly justified.

As was explained in Section “The Constructive Nature of Memory”, the phenomenon of boundary extension (an error of commission) tends to be remarkably accurate, so much so that Michaelian (2016b, p. 123) claims that “boundary extension need not reduce the reliability of remembering.” And since Michaelian endorses reliabilism about justification, it follows that, by his own lights, there are mnemonic confabulations that meet the justification condition.

Hirstein (2005, p. 207) follows Goldman (1998) in demanding that a justified belief not only be based in a reliable belief-forming process but also that and there be no reliable or conditionally reliable process available to the subject which, had it been used by the subject in addition to the process actually used, would have resulted in his not believing p. Given this version of reliabilism, it is highly unlikely that there could be confabulations that meet the justification condition.

The two advocates of the epistemic theory of confabulation, Hirstein and Michaelian endorse reliabilism about justification. Yet it is possible to combine the epistemic theory of confabulation also with other accounts of justification. According to the coherence theory of justification, for example, a belief is justified in virtue of belonging to a coherent system of belief. And for a system of beliefs to be coherent, the beliefs that make up that system must cohere with one another^23^. Typically, coherence is taken to involve three components: logical consistency, explanatory relations, and various inductive (non-explanatory) relations. As was explained in Section “Confabulation without Falsehood”, confabulations can be “coherent, internally consistent, and relatively commonplace” (Moscovitch, 1995, 226–227). Given that the justification of a belief consists in its coherence with other beliefs in the system and given that confabulations can be coherent it follows that confabulations can meet the coherentist justification condition. But if confabulations can count as coherentistically justified, then the epistemic account fails in its attempt to identify the feature that sets confabulations apart from memories.

Coherentism and reliabilism are not the only accounts of epistemic justification whereupon confabulations can count as justified. Other cases in point are the causal theory of knowledge (Goldman, 1967), the failability account of knowledge (Hetherington, 2001), deontological conceptions of epistemic justification (Chisholm, 1977), as well as certain forms of evidentialism (Conee and Feldman, 2004). Yet discussing these theories of epistemic justification would take us too far afield.

## The causal theory of memory

We have seen that there are compelling reasons to reject the two extant accounts of mnemonic confabulation as “false memory” and as ill-grounded memory. And since there can be confabulations that are true *and* justified it does not help to define confabulations as memory beliefs that are *either* false *or* ill-grounded^24^. Where does all this leave us? Are we forced to conclude that confabulation is “an ill-defined symptom” (Victor et al., 1989, p. 43) or “merely a polite term for plain lying” (Whitlock, 1981, p. 213)? No. The point of the paper is to propose a novel account of confabulation whereupon the defining characteristic of confabulation vis-à-vis genuine memory is not that it is false or that it is ill-grounded but that it lacks the appropriate causal history. In this section I set forth a version of the causal theory of memory^25^. In the following section, I argue that confabulations fail to satisfy the causal condition on remembering.

Intuitively, to remember something is different both from learning it anew and from verdically confabulating it. A claim to remember something implies not merely that the subject represented it in the past, but that her current representation is in some way due to, that it comes about because of, her past representation. A theory of memory must therefore devise a *connection condition* that ensures that the memory content is retained rather than relearned or fabricated. The connection condition states that, to remember a proposition, not only must it have been represented before, but the present representation must be suitably connected to the past representation.

The connection conditions proposed in the literature fall into three categories: the evidential retention theory, the simple retention theory, and the causal theory. The *causal theory* states that to remember that p one's present representation must stand in an appropriate causal relation to one's past representation that p^*^, where p is identical with, or sufficiently similar to, p^*^^26^. The crucial issue is, of course, to determine what should count as an *appropriate* causal connection. I will return to this issue below. The main competitors with the causal theory are the evidential and the simple retention theory. Proponents of the *simple retention theory* such as Squires (1969) hold that for a past and present representation to be memory-related, what is required is merely that by virtue of having had a particular past representation, one acquired an ability or disposition that one retained and now exercises by occupying the present representation; there need not be a causal connection between the past and present representation. According to the *evidential retention theory* first proposed by Naylor (1971), for a piece of knowledge to qualify as a memory its justificatory factors (evidence, grounds, reasons) must be the same as those supporting the original piece of knowledge that has been retained. To remember that p you must know that p, you must have known that p in the past, and your grounds for believing p in the past must be the same as your grounds for presently believing that p. On this view, retaining knowledge involves not only retaining known propositions but also supporting reasons.

Three reasons speak in favor of the causal theory of memory and against competing theories. First, the simple retention theory states that the retention process is not of a causal kind but it does not give us a lead as to the kind of process responsible for the retention of the ability to represent a proposition. The causal theory has an explanatory advantage over the simple theory in that it gives an answer to the question of what kind of process makes memory retention possible. Second, unlike the evidential retention theory, the causal theory is not committed to the problematic thesis that memory implies epistemic justification (see Section “Against the Epistemic Theory of Confabulation”). Third, the causal theory of memory provides a better explanation of the truth of the commonsensical counterfactual “If S had not represented at t_1_ that p^*^ he wouldn't represent at t_2_ that p” than either the simple or the evidential retention theory.

A more recent rival for the causal theory of memory is (non-causal) *simulationism* (Michaelian, 2016b)^27^. Simulationism states that is in episodic remembering the subject draws on information acquired during experience of past events to construct a simulation of a target event from his personal past. The proper functioning of the memory system is defined in terms of three conditions—accuracy, internality, and reliability. The internality condition states that the remembering subject must himself contribute either retained or generated content to the memory representation (Michaelian, 2016b, p. 10). The reliability condition states that for the memory system to function properly it must have the tendency to produce mostly accurate representations. And the combination of the three conditions is meant to make the causal condition superfluous. As Michaelian (2016b, p. 7) declares: “Instead of defining the proper functioning of the system in terms of retention of information, they define it directly in terms of reliability. …[O]nce a reliability condition is added to the theory, the causal condition itself is no longer necessary.”

In response to the simulationist challenge to the causal theory of memory I want to make three points. First, insofar as the content of the memory representation is retained (not constructed), the question arises as to what is involved in the process of retaining content. The simulationist theory, like the simple retention theory, does not provide an answer. I submit that if proponents of simultanionism tried to spell out the process underlying the retention of content they would ultimately fall back on the causal theory of memory. Second, simulationism defines memory in terms of the reliable production of accurate representations. But how does our memory system manage to reliably produce accurate representations? If this question is not answered in terms of a causal process connecting the past and present representation, then, as far as I can see, we are left with a picture whereupon there is a remarkable correlation between our memory representations and past events but nothing to explain the correlation. Once again, the causal theory of memory has a clear explanatory advantage over simulationism in that it explains the process underlying the remarkable correlation.

Not just any sort of causal connection constitutes memory; some causal chains are not of the appropriate sort; they are deviant. But what counts as an *appropriate* causal connection between a past and present representation? Intuitively the past representation must be stored in a memory trace which represents the original event and provides a causal link between the original episode and the subject's ability to remember the event. When a state of remembering is the joint product of a trace and a retrieval cue, the trace must be an indispensable part of the jointly sufficient condition responsible for the production of the memory. Another way of making the point is to say that the memory trace must be at least an insufficient but non-redundant factor of an unnecessary but sufficient condition for the state of seeming to remember. Mackie (1965) calls such a factor an *inus condition*. Characterizing the causal dependence of a state of remembering on a trace in terms of *inus conditions* allows us to distinguish remembering something upon being prompted from merely repeating back the prompt itself.

There is a crucial difference between the causal dependence of memories on traces, on the one hand, and the causal dependence of memories on past representations, on the other. The dependence of memories on traces vis-à-vis prompts is best analyzed in terms of necessary and sufficient conditions. The causal dependence of a memory on the corresponding past representation is best characterized in terms of a counterfactual conditional: If S hadn't represented at t_1_ that p^*^ she wouldn't represent at t_2_ that p. This condition allows us to correctly classify abnormal cases in which a past representation matches a state of seeming to remember by triggering some one-off random causal mechanism which produces an ostensible memory whose content has been previously entertained by the subject.

To motivate the counterfactual conditional above consider the following case. Due to a severe depression, Jane is convinced that no one likes her. Whenever she meets someone who acts friendly toward her, the experience gets transformed in her memory, so that, later on, it seems to her as if the person had acted unfriendly toward her. Jane is unaware of her memory's bias. At t_1_, Jane has an encounter with Jennifer who openly displays her dislike for Jane. At t_2_, Jane seems to remember that Jennifer disliked her when they met at t_1_. Even though Jane's memory claim is true and there is a causal relation between her experience at t_1_ and her ostensible memory of that experience at t_2_ she does not remember that Jennifer disliked her. The reason Jane doesn't remember is that, all things being equal, if, at the time, Jane had thought that Jennifer likes her, she would still believe at t_2_ that Jennifer had acted in an unfriendly manner. It is just a matter of luck that the content of Jane's ostensible memory matches that of her past representation. Yet intuitively, to remember something, the correspondence between the contents of one's past and present representations may not be entirely by accident. For a state of seeming to remember to be memory-related to a past representation it has to be the case that, if the past representation had been different, then one would not occupy the very state of seeming to remember that one does occupy.

## A causal theory of mnemonic confabulation

The defining characteristic of mnemic confabulation vis-à-vis genuine memory is that the state of seeming to remember fails to counterfactually depend on the corresponding past representation, provided there is a past representation. In some cases, the confabulatory state purports to represent a particular past representation but there is no past representation. In other cases, there *is* a past representation and confabulation occurs because the following counterfactual conditional is not satisfied: if the past representation had been different, it would have caused a different state of seeming to remember to match the different past representation. It is the hallmark of confabulation that any match between the contents of the past and present representations is nothing but a lucky accident. In genuine remembering, however, just as the actual past representation causes a matching state of seeming to remember, so likewise would alternative past representations. Different past representations would produce different states of seeming to remember.

The causal theory of confabulation has been occasionally mentioned in the literature but it has never been elaborated nor defended against alternative conceptions. Martin and Deutscher (1966, p. 173–175) note in their seminal paper “Remembering” that the causal condition on remembering guards against the possibility of veridical confabulation^28^. They consider the following case. Kent experiences a car accident. His memory of the accident is wiped out by a second car accident that Kent is involved in shortly after the first accident. At this point, he no longer remembers the first accident. Again, shortly afterwards, a hypnotist produces in Kent the belief that he had been in a car accident at a certain time and place. By sheer coincidence the hypnotist's description of the accident matches Kent's first accident. After having been hypnotized, Kent correctly believes that he has been in a car accident of a certain kind at a certain time and place. Notwithstanding the fact that Kent is describing correctly the first accident Martin and Deutscher (1966, p. 174) claim that he does not remember because “his recounting of the first accident is not due, even in part, to his observing it.” I agree with Martin and Deutscher's treatment of veridical confabulation. But unlike them I employ the causal condition not only to rule out veridical but not falsidical confabulation.

Besides Martin and Deutscher, the only other proponent of a causal theory of confabulation is Robins. In (2017a) Robins presupposes, but does not develop, a causal theory of confabulation when she argues that many constructive theories of memory cannot account for confabulations because they deny the existence of a causal connection in cases of genuine remembering and so can't distinguish real memories from confabulations where the connection is missing (Robins, 2017a). The crux with Robin's account, however, is that it cannot account for veridical confabulation. For a state as of remembering to qualify as confabulation, according to Robins, it is not enough that the causal condition is violated; the content of the state also needs to be false^29^. But as we have seen in Section “Confabulation without Falsehood”, confabulations need not be false.

The main idea of the causal theory of confabulation is that a subject is confabulating when her state of seeming to remember fails to counterfactually depend on the corresponding past representation, provided there is a past representation. This definition of confabulation raises the question of whether it matters by which means the counterfactual dependence is established^30^. To illustrate the latter problem, consider a version of Martin and Deutscher's car accident example. This time, however, Kent's describing correctly the first accident is not a chance event. Instead the hypnotist would not produce in Kent the belief that he had been in a car accident at a certain time and place unless this belief was true. In this case, the causal dependence clause seems to hold, even though it is debatable whether it is a case of genuine remembering. The assessment of this case depends on how wide a range of possible changes a person's ostensible memory must depend on. Borrowing a term from Nozick (1981, p. 178) we can say that an ostensible memory *tracks* a past representation when a wide range of possible changes of the past representation brings about corresponding changes of the ostensible memory. I am inclined to think that deviant causal chains *are* compatible with remembering as long as the state of seeming to remember tracks the corresponding past representation. Yet the plausibility of the causal theory of confabulation is independent on where one stands with respect to the issue of deviant causal chains.

The causal theory of confabulation delivers the right verdict in the case of veridical and justified confabulation. For just because a state of seeming to remember is true by coincidence doesn't mean that it counterfactually depends on the corresponding past representation. And likewise whether a state of seeming to remember is, say, coherentistically justified has no bearing on whether it counterfactually depends on the corresponding past representation. That is why the causal theory of confabulation allows us to eliminate verdicial and well-grounded confabulations from the ranks of genuine memory.

## Confabulating, misremembering, and relearning

Michaelian (2016b) has recently launched an interesting attack on the causal theory of confabulation. He grants that the causal theory of confabulation allows us to distinguish between remembering and veridical confabulation, on the one hand, and misremembering and falsidical confabulation, on the other. What the causal theory of confabulation does not allow us to do, according to Michaelian, is to differentiate between confabulation, one the one hand, and relearning and misremembering, on the other.

Michaelian treats relearning, confabulation, and misremembering as distinct kinds of “memory errors.” Relearning “occurs in certain cases in which the subject's memory of an event depends entirely on an external prompt” (Michaelian, 2016b, p. 3). While relearning is about “lack of internality,” confabulation is said to be about “lack of reliability” (see Section “Against the Epistemic Theory of Confabulation”). Misremembering “occurs when the reliability condition is met but the accuracy condition is not” (Michaelian, 2016b, p. 9).

With the distinction between relearning, confabulation and misremembering in place, Michaelian argues that the causal theory of confabulation has the consequence of annihilating the distinction between veridical confabulation and relearning. The causal approach, he claims, “does not distinguish between veridical confabulation and relearning, neither of which involves a trace connection” (Michaelian, 2016b, p. 4–5). Presumably the idea is that, from the point of view of the causal theory of memory, there is no difference between a case of relearning where there are no memory traces whatsoever (lack of internality) and a case of veridical confabulation where there may be some memory traces but where they fail to play the appropriate role for the counterfactual dependence of the ostensible memory state on the corresponding past representation. And besides conflating veridical confabulation and relearning, the causal theory also conflates falsidical confabulation and misremembering. Michaelian writes:

[T] he causalist will …have difficulty distinguishing between misremembering and falsidical confabulation. Because he sees misremembering as being characterized by reliability and inaccuracy and falsidical confabulation as being characterized by unreliability and inaccuracy (Michaelian, 2016b, p. 10).

The point of this section is to show that Michaelian's objections to the causal theory of confabulation do not hold up to scrutiny. I will make three points.

First, it is a mistake to include relearning in a taxonomy of memory errors. Relearning occurs when one learns information, forgets it, and then re-acquires it either from the same or a different source (Robins, 2017b, p. 82) The availability of various sources (diaries, photographs etc.) about some past event, together with the elusiveness of memory, creates the possibility that one reacquires, rather than retains, information about the event.

Relearning is clearly different from remembering, but this does not mean that relearning is a memory *error*. Relearning is typically preceded by *forgetting* which may or may not be regarded as a memory error^31^. And relearning is sometimes accompanied by a *source-monitoring error* which is a type of memory error where the source of a memory is incorrectly attributed to some specific recollected experience (Johnson et al., 1993). For instance, you may relearn about a current event from the local news, forget the event, relearn about the event from a friend, but later report having learned about it on the local news (where you originally acquired the information), thus reflecting an incorrect source attribution. So even though relearning may be preceded by a memory error (forgetting) and may be accompanied by a memory error (source-monitoring error) it is not itself a memory error. Michaelian is aware that

relearning…is not among the memory errors standardly studied by psychologists. But relearning is clearly closely related to remembering, and, while it is not natural to view relearning as an error if the subject is aware that he is relearning, the same thing goes for the other errors discussed here—in the cases with which we are concerned, if it is assumed that the subject takes himself to be remembering. Hence it is appropriate to include it in a taxonomy of memory errors^32^.

What Michaelian seems to be saying is that relearning is a memory error if the subject mistakenly takes himself to be remembering. But even if we accept this claim, what is problematic about relearning is the erroneous source monitoring, not the re-acquisition of information. Any way you look at it, relearning is not a memory error.

Second, Michaelian claims that the causal theory conflates veridical confabulation and relearning since “neither of which involves a trace connection.” The underlying assumption is that the causal theory can only distinguish relearning from veridical confabulation by means of the severed causal connection. And since the causal connection is severed in either case, there is nothing to distinguish veridical confabulation and relearning, or so Michaelian thinks. But this is simply not the case. There are other ways for the causal theorist to distinguish veridical confabulation and relearning. For starters, relearning, unlike confabulation, involves the re-acquisition of information. Another difference has to do with the attitude of the subject. We saw that confabulations are taken by the subject to be genuine memories. Yet in ordinary cases of relearning the subject does not falsely take himself to be remembering.

Third, Michaelian claims that the causal theory conflates misremembering and falsidical confabulation since both are seen “as being characterized by inaccuracy.” Even if we were to accept Michaelian's definition of misremembering as reliable but false^33^, the criticism does not hold, for there are other ways for the causal theorist to distinguish misremembering and falsidical confabulation. As Michaelian himself notes, the causal theorist can point to the satisfaction of the reliability condition as a *differentia specifica* of misremembering vis-à-vis falsidical confabulation. Furthermore, people who confabulate stories are often very confident in their “memories” even after being shown contradicting evidence; this is not usually the case with ordinary cases of misremembering.

## Considerations of clinical utility

“Confabulation” is neither an ordinary, everyday term (such as “memory” and “forgetting”), nor a technical philosophical term (such as “justification” and “knowledge”) but instead a medical term (such as “brain lesion” and “vitamin B1 deficiency”). Given that “confabulation” is a medical term, we should expect the criteria for confabulation to be verifiable in a clinical setting. But the worry is that the causal theory of confabulation has less strong ties to clinical practice and to research in cognitive sciences than, say, the epistemic theory of confabulation. For while it appears to be possible to verify whether a patient's memory belief is or isn't well-grounded it does not appear to be possible to verify whether the process that gives rise to a patient's memory belief satisfies the counterfactual dependence clause discussed in Section “The Causal Theory of Memory”; or so a critic might argue. Two points I think need to be made in response to the concern that the causal theory of confabulation does not improve diagnosis.

As was explained in Section “Against the Epistemic Theory of Confabulation”, the epistemic theory of confabulation comes in two flavors. Michaelian defends an externalist-reliabilist reading of the epistemic theory while Hirstein's notion of justification combines both internalist and externalist elements. Let us start with Hirstein.

Hirstein (2005, p. 204) endorses a hybrid theory of justification that involves both internal and external features. The external feature of justification consists in the absence of brain damage and the internal features consists in the coherence among the subject's mental states as well as in their likeness to truth. Hirstein (2005, p. 204) writes: “Confabulators often seem to believe that their confabulations are justified by other states, by alleged memories or perceptions, but typically those states are ill-grounded also.” So, according to Hirstein, it is not enough to ensure that one's thoughts are coherent; the thoughts must also well-grounded in the sense of having a high degree of probability of truth. Hirstein lists a high degree of probability of truth among the *internal* features of justification because he assumes that a healthy subject has “the ability to assess the probability of a thought being true” (2005, p. 208).

But how can we tell whether our seeming memory experiences are indeed representations of the past? An obvious suggestion is that we can know that our ostensible memories are true by checking them against diaries, photographs, testimony and the like. The problem with this kind of evidence, however, is that it begs the question at issue—whether one's ostensible memory supplies knowledge—in that the employment of this evidence assumes the trustworthiness of some memory (one's own or someone else's). To drive this point home, suppose that you seem to remember putting a key in the drawer. You open the drawer, and there is the key. Does this not confirm your memory experience? No, for the key might be in the drawer without your having put it there. Then suppose that you try to confirm your memory experience of having put the key in the drawer by asking your friend whether she saw you put the key there. If your friend answers in the affirmative, does this justify your memory experience? No, for your friend's (ostensible) memory experience might be just as much a product of the fancy as your own. The problem of verifying ostensible memory is only pushed from you to your friend. The upshot is that any attempt to confirm the validity of memory experiences is circular. There is no non-question begging way of determining that one's ostensible memories are true. No one memory can be validated without relying on other memories (cf. Bernecker, 2008, 97–104). The upshot is that Hirstein's internalist justification condition whereupon we need to “assess the probability of a thought being true” is difficult to use in clinical practice.

Next consider Michaelian's externalist–reliabilist reading of the epistemic theory of confabulation. Traditionally knowledge has been defined as justified true belief. According to reliabilism, the justification condition should be replaced by the condition that the true belief was generated by a reliable process. Knowledge is reliably produced true belief. Michaelian explains that the notion of reliability underlying his simulationist theory is the modal notion as opposed to the statistical or probabilistic notion (Michaelian, 2016b, p. 6; cf. Michaelian, 2016a, p. 140–142). According to the *probabilistic* notion, a belief-forming process is reliable if and only if the conditional probability to acquire a true belief on the basis of the process is greater than a certain value. In normal cases of reliable processes, we can expect the value to be >0.5. According to the *modal* notion, a belief-forming process is reliable if and only if in the actual world and in some suitably qualified possible worlds the number of true beliefs to the number of false beliefs resulting from the process is above a certain value. The value need not be a precise one and might vary with context. What is important to realize is that the modal notion of reliability is couched in terms of counterfactuals, i.e., subjunctive conditional statements whose antecedent states a counter-to-fact situation.

Given the modal notion of reliability, it doesn't seem to be possible to empirically determine whether a given process is reliable. Devising an experiment to figure out that whether a subject in a some possible world would acquire more true than false beliefs on the basis of some process seems to be just as hopeless as determining whether a subject would represent at t_2_ that p even if (counter to fact) she had not represented at t_1_ that p^*^. Since it doesn't seem to be possible to empirically test counterfactuals it is common to think that they “cannot be relied upon and fruitfully used in empirical-practical domains such as medicine” (Sadegh-Zadeh, 2015, p. 250). But maybe this is an overhasty conclusion. Reconsider the counterfactual notion of causation. There is a clear connection between the notion of counterfactual dependence and the notion of influence and manipulability: if event x depends on event y, then x is influenced or manipulated by y^34^. The manipulability view of causation is common in the empirical sciences: x is the cause of y if changing x changes y, i.e., if we can manipulate y by manipulating x. And given the connection between counterfactual dependence and manipulability it seems to be possible to interpret experiments that test for the presence of influence and manipulability as testing for the presence of counterfactual dependence (cf. Winship and Morgan, 1999).

We may conclude that considerations of clinical utility cannot be used to adjudicate between causal and epistemic accounts of confabulation. The reason is that both Michaelian's reliabilist account of confabulation and my own causal account of confabulation operate with counterfactual conditionals. We also saw that there may be indirect ways of testing a statement to the effect that two events are counterfactually dependent on one another.

## Conclusion

In sum, what defines confabulations vis-à-vis genuine memories is not that they are false or lack epistemic justification but that they fail to be suitably causally connected to the corresponding past representations, either because there are no corresponding past representations or because the causal connection has been severed^35^.

## Author contributions

The author confirms being the sole contributor of this work and approved it for publication.

### Conflict of interest statement

The author declares that the research was conducted in the absence of any commercial or financial relationships that could be construed as a potential conflict of interest.

## Footnotes

^1^The technical term “confabulation” was coined by Karl Bonhoeffer, Arnold Pick, and Carl Wernicke in the early 1900s and was applied to patients with Korsakoff's syndrome (Berrios, 1998). “Fabrication,” “false memory,” and “pseudo reminiscence” are sometimes used to mean the same thing as “confabulation.”

^2^Gilboa et al. (2006, p. 1411). According to Carruthers (2009), it is our mindreading capacity that is responsible for all kinds of confabulations, not only mnemonic confabulations.

^3^I am grateful to a reviewer for pressing me on this point.

^4^I take the term “propositional memory” to refer to any substituend of the schema “S remembers that p,” irrespective of whether “p” refers to something one has personally experienced, and irrespective of whether the memory content consists merely of the proposition p or whether, in addition, it includes images and qualia. In other words, propositional memories can be both episodic and semantic.

^5^I don't mean to suggest that Plato and Aristotle endorse the xerox model of memory. The wax tablet metaphor is propounded to clarify the relationship between perceiving and knowing without implying that this is all that is involved in memorizing and recall. Nevertheless, the metaphor illustrates the dangers of being captured by the attractions of a picture. Most classical and medieval writers seem to have been satisfied with the wax tablet as a model of memory.

^6^This distinction does not align with the distinction between omission errors and commission errors. Schacter (2001) distinguishes three kinds of omission errors—transience, absent-mindedness, blocking —and four kinds of commission errors—misattribution, suggestibility, bias, and persistence. Omission errors may or may not change the truth-values of the encoded contents.

^7^The concept of leveling has its roots in Gestalt psychology. Gestalt psychologists contrasted leveling with sharpening, in which a person exaggerates selected characteristics of the encoded content. See Kellogg (2012, p. 158).

^8^Leveling may not preserve the falsity of the encoded content. Suppose you believed that there is a bird in the tree but it is in fact a cat. If what you remember is that there was an animal in the tree then leveling has transformed a false into a true belief.

^9^Hubbard et al. (2010), Michaelian (2011b, p. 325–326), and Michaelian (2011a, p. 123). Boundary extension is taken to be a consequence of the fact that information about the likely layout of the scene is automatically retrieved and then incorporated into the memory of the scene. So the content of the memory is a combination of the stored content and some additional content.

^10^The notion of relevant entailment is meant to ensure that the content of the present propositional attitude is not on a completely different topic than the content of the past propositional attitude. According to Anderson and Belnap (1975), p is relevant to q if and only if p could be used in a deductive argument of q from p. They reject, for instance, p ⊃ (q ⊃ q) because p may be irrelevant to (q ⊃ q) in the sense that p is not used in arriving to (q ⊃ q). To infer q from p it is necessary that p and q have some common meaning content. Since Anderson and Belnap hold that in propositional logic commonality of meaning is carried by commonality of propositional variables, they conclude that p and q should share at least one propositional variable.

^11^Bernstein and Loftus (2009, p. 373). Given that knowledge implies truth, “what we know about the world” is true.

^12^Conway and Loveday (2015, p. 580). “The healthy human brain is not a veridical recorder of events but rather a meaning machine that fills gaps, rearranges time and space, delays conscious experience, and generates false explanations via available cultural theories. To confabulate is human” (Wheatley, 2009, p. 219). “Commonly, memories can be true without being perfectly accurate or verbatim reports of past events” (Sutton, 2003, p. 146).

^13^Talland (1965, p. 49) also characterizes confabulations as “more or less coherent and internally consistent.” And Van Damme and d'Ydewalle (1965, p. 212) declare: “As [momentary] confabulation is considered coherent and internally consistent, and mainly comprises true memories being displaced in time and context, it closely resembles the type of memory distortion we all sometimes produce.”

^14^Dalla Barba (1993, p. 10), DeLuca (2001, p. 121), and Moscovitch (1989, p. 135–136).

^15^For the fallibilism/infallibilism distinction see Dougherty (2011) and Hetherington (2017).

^16^Although the degree of conviction manifested in confabulation varies (DeLuca, 2001, p. 121; Schnider, 2008, p. 70–71), it is usually thought that patients are typically sincere in their confabulations, and are unaware of their inaccuracies (Johnson et al., 2000, p. 383; Moscovitch, 1995, p. 226; Ramachandran, 1995, p. 28–32).

^17^I am assuming the standard conception of knowledge whereby knowledge involves belief. Williamson (2000) and others have suggested reversing the order of explanation between knowledge and belief: instead of analyzing knowledge in terms of belief, the concept of knowledge should be used to elucidate the concept of belief.

^18^Hirstein (2005, p. 225–226) argues that the word ‘should’ is to be interpreted differently if the person has brain damage, or has a normal brain. If the person has brain damage, Hirstein claims that ‘should’ is to be interpreted in a functional sense (as in “the motor should work, it's got power”). If the person has a normal brain, ‘should’ is, additionally, to be interpreted in a normative sense, in which the person is held responsible for failing to correct the thought, or at least that he does not have knowledge. However, in both types of cases, should implies can. See Sinnott-Armstrong (1984). My discussion of Hirstein's condition (v) follows Bortolotti and Cox (2009, p. 956–961).

^19^The interpretation whereupon the subjects in Nisbett's and Wilson's experiment are confabulating is due to Carruthers (2005, p. 142–147). To be fair, it should be mentioned that Hirstein (2005, p. 189) is aware that his definition of confabulation does not provide necessary conditions.

^20^The claim that genuine memories may be unjustified flies in the face of the widespread *epistemic theory of memory* whereby remembering that p implies knowing that p. If memory implies knowledge and if knowledge implies justification, it follows (by transitivity of implication) that memory implies belief, truth, and justification. The epistemic theory of memory is endorsed, among others, by Anscombe (1981), Ayer (1956, p. 138, 147–148), Dretske and Yourgrau (1983), Huemer (1999), Pollock and Cruz (1999, p. 46–48), Shoemaker (2003, p. 43), and Williamson (2000, p. 37–38).

^21^The value of the index in the subscript to “t” determines whether the time referred to is in the past or the present: the relatively biggest number indicates the present. So here “t_2_” is the present and “t_1_” is the past.

^22^Cf. Bernecker (2010, p. 71–83, 2011, p. 112–116). For dissenting views see Adams (2011) and Moon (2013).

^23^For a survey of coherentist theories of memorial justification see Olsson (2017).

^24^According to Sullivan-Bissett (2015, p. 551), it is a common feature of confabulatory explanations that they are false or ill-grounded. “Confabulatory explanations are epistemically faulty. Generally speaking, they are *false* explanations…But even when confabulatory explanations are not false, they are epistemically poor in other respects. A key epistemic feature of confabulations then is that they are *ill-grounded* or *poorly supported by evidence*.”

^25^The causal theory of memory sketched in this section is developed in Bernecker (2010, chs. 4, 5).

^26^The classic formulation of the causal theory of memory is due to Martin and Deutscher (1966). Among the critics of the causal theory are Shope (1973) and Zemach (1983).

^27^Some versions of simulationism are compatible with the existence of a causal connection between the past and present representation (DeBrigard, 2014). Here, I focus on Michaelian's version of simulationism which purports to do without the retention condition.

^28^This has been noted by Michaelian (2016b, p. 4) and Robins (2016b, p. 2997).

^29^Robins (2016a, p. 445) writes: “Misremembering occurs when the first retention condition is met, but the second accuracy condition is not. When neither condition is met, the result is confabulation.”

^30^An analogous problem faces Lewis's (1986) causal theory of hallucination.

^31^Michaelian (2011a) argues that a certain pattern of forgetting is necessary if our memory is to perform its function well.

^32^Michaelian (2016b, p. 4). Robins (2016a, p. 446, n.17) also classifies relearning as a memory error.

^33^For an alternative definition of misremembering see Robins position cited in footnote 29.

^34^Here, I follow Paul (2011, p. 170–171).

^35^Ancestors of this paper were presented at a conference on Medical Knowledge in a Social World held at the University of California, Irvine, March 28–29, 2016 and at the 6th International Conference on Memory held in Budapest, Hungary, July 17–22, 2016. For comments on previous drafts I am grateful to Jordi Fernández, Kourken Michaelian, Cailin O'Connor, Maura Priest, Sarah K. Robins, Markus Werning, and three reviewers for this journal.
